# Editorial: Women in anti-doping sciences & integrity in sport: 2021/22

**DOI:** 10.3389/fspor.2023.1248720

**Published:** 2023-07-11

**Authors:** Andrea Petróczi, Kim Nolte, Angela Jo-Anne Schneider

**Affiliations:** ^1^ School of Life Sciences, Pharmacy and Chemistry, Faculty of Health, Science, Social Care and Education, Kingston University, Kingston upon Thames, United Kingdom; ^2^ Institute of Health Promotion and Sport Sciences, Faculty of Education and Psychology, Eötvös Loránd University, Budapest, Hungary; ^3^ Department of Physiology, Division of Biokinetics and Sport Science, Faculty of Health Sciences, School of Medicine, University of Pretoria, Pretoria, South Africa; ^4^ School of Kinesiology, International Centre for Olympic Studies, Western University, London, ON, Canada

**Keywords:** anti-doping, integrity, research output, impact, gender difference

**Editorial on the Research Topic** Women in anti-doping sciences & integrity in sport: 2021/22

To address the frequently observed gender inequality in research, considerable efforts have been made on international and national levels via informing policymakers, creating more equal opportunities for women scientists, and improved governance (1–3). Calls have been made to pay attention to the impact of gender disparities in funding allocations (4), impact assessment (5), peer reviews of grant application (6) and publications (7). Evidence also indicates that women's qualifications and ability are underestimated (8, 9) leading to different outcome expectations (10) and women receiving less credit (11, 12), their outputs being more critically scrutinised (13) or held to higher standards (14).

**Figure 1:**
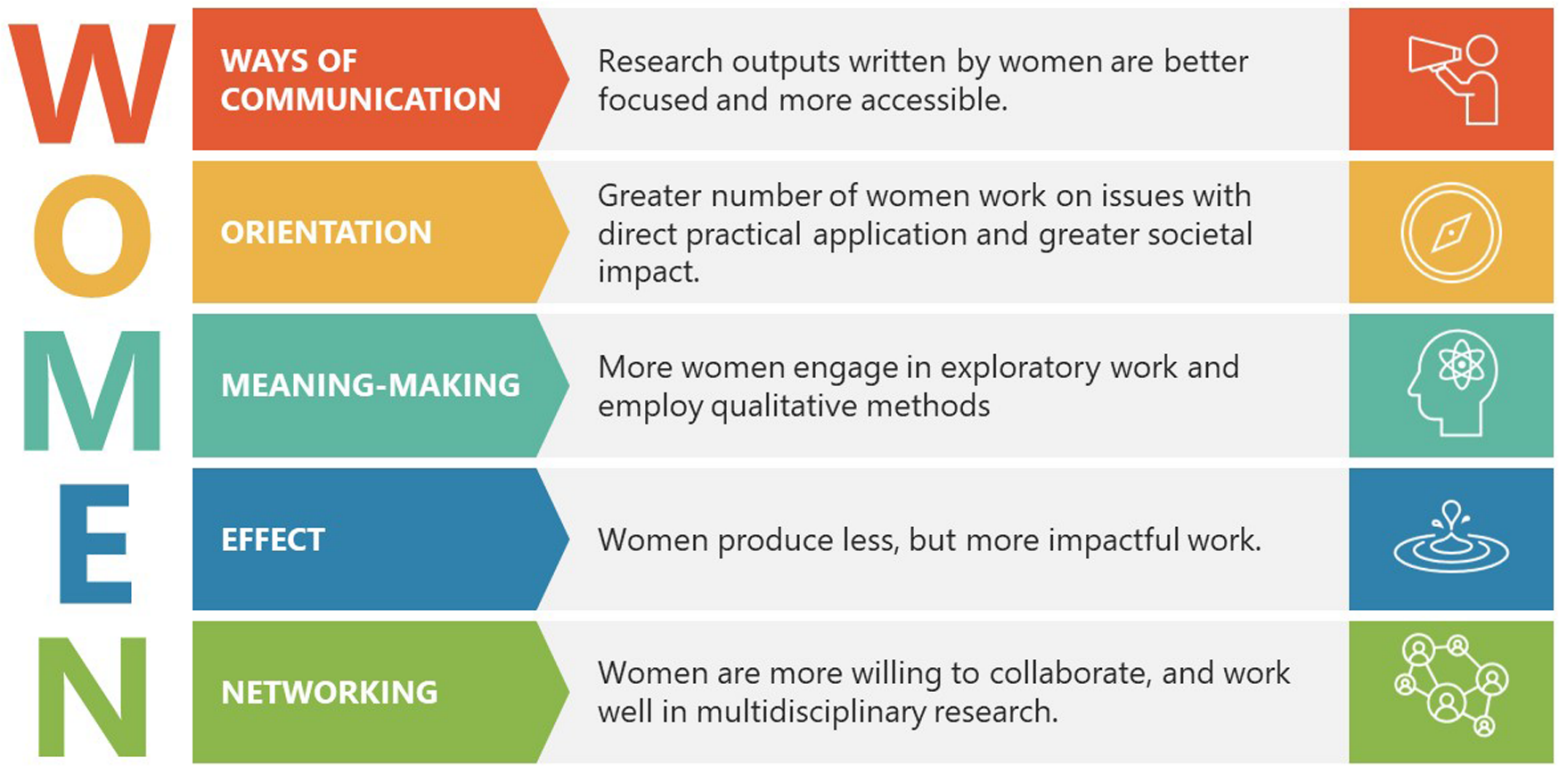
Valuable characteristics of women's research and research outputs.

These are just examples of where gender bias against women exists. All in all, there is ample evidence showing that research is an unwelcoming field for women despite the fact that they can bring unique contributions to the table by creating impact with fewer outputs and less money, and a natural orientation toward making societal impact via research focus, and a communication ‘style’ coined as the female scientific voice that suits users of scientific knowledge outside academia better (Figure 1). Despite more modest self-presentation (15), lower number of outputs (16) and patents (17), women are equal if not better than male researchers when it comes to research impact (18, 19). This might be explained by the research focus where women are more likely to tackle societal issues (20), and gender differences in definition and attitude toward impact (21) in which women see impact in societal context (i.e., achieving social justice and equality) as opposed to male researchers who tend to focus on academic impact, accountability and responsibility toward society. There are stronger tendencies for women to engage in meaning-making research via exploratory and qualitative investigation (22). Lastly, it has been observed that women are more willing to collaborate, and when they do, they tend to ‘pair up’ (23), work with a smaller number of collborations but there is no agreement whether women have more transient (24) or stable (25) collaborations.

**Figure 2:**
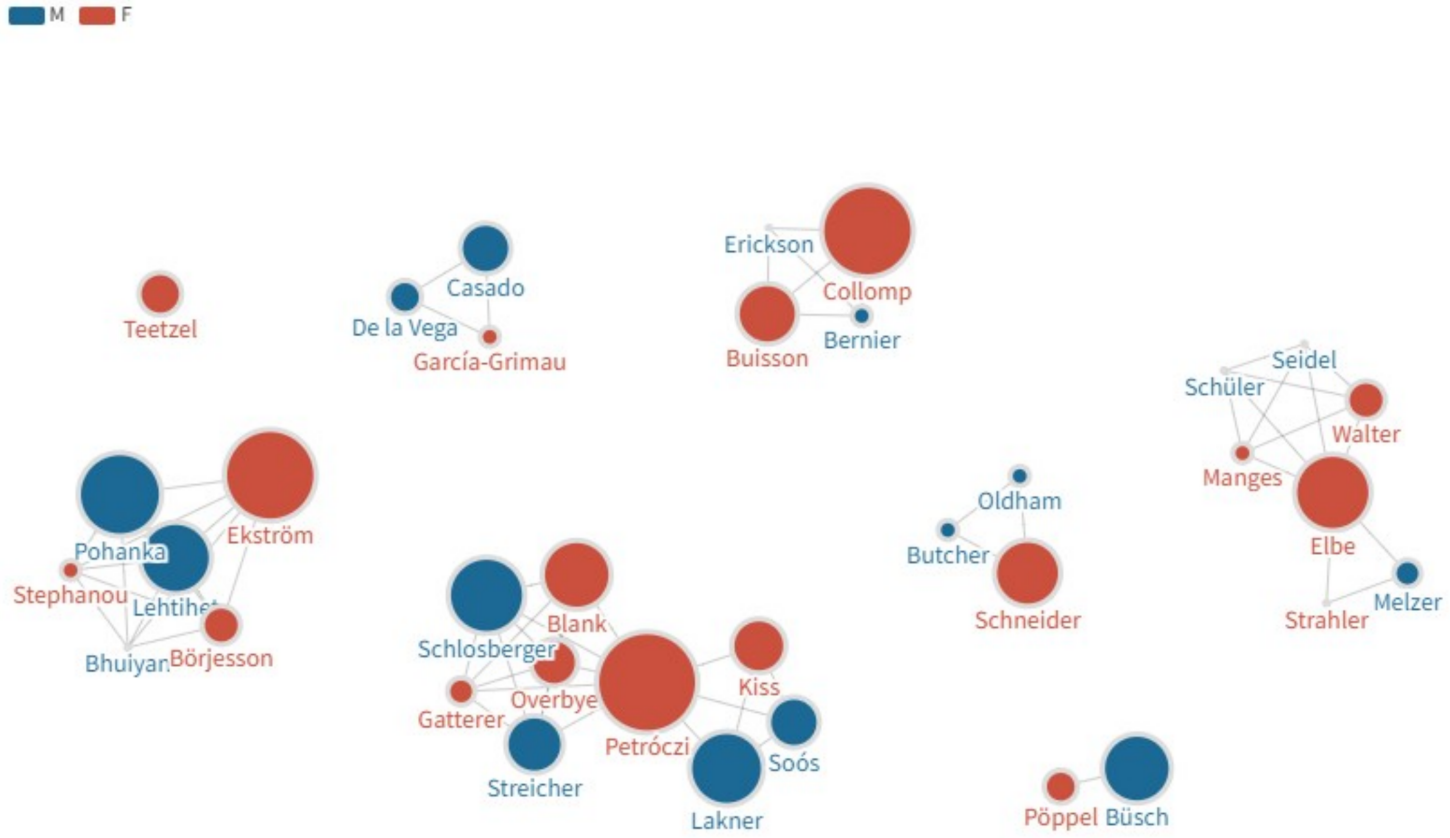
Authorship network map. Colour denotes gender, size of the nodes corresponds to the authors’ H-indices based on Web of Science in 2022 December). Authorship positions are displayed in the interactive version of this network map: https://public.flourish.studio/visualisation/14246812/. Move the cursor above the nodes to see the authorship positions on the interactive map.

The collection in this special issue features 10 papers spearheaded by women researchers featuring 35 unique authors in total, of which almost half (48.6%) are male (Figure 2, https://public.flourish.studio/visualisation/14246812/). These papers include three studies on gender differences focusing on the prevalence of prohibited substances and methods by female athletes (Collomp et al.), research standing and strength of women scientists in anti-doping (Kiss et al.), and women’s sport as a protected category (Schneider et al.). The remaining seven papers cover non-gender related topics but authored by women as first (Schneider et al.) or corresponding author (Lehtihet et al.). Seven papers feature original research (Blank et al., Collomp et al., García-Grimau et al., Kiss et al., Lehtihet et al., Schneider et al.), followed by two brief research reports (Melzer et al., Pöppel and Büsch) and an opinion piece (Teetzel). Only one paper (Lehtihet et al.) falls within the natural sciences domain, with a focus on detecting Anabolic Androgenic Steroid use. Subject areas of the submitted studies shows a diverse picture both in terms of topics and methodology. Four papers focused more on societal issues such as prevalence (Collomp et al.), sanctioning young athletes (Teetzel), women’s sport (Schneider et al.), or the role women researchers played in generating anti-doping knowledge to date (Kiss et al.). Others concentrate on practical aspects such improving doping testing (Lehtihet et al.), values-based anti-doping education programme for adolescent athletes (Manges et al.), need for tailored education programme for young athletes (Pöppel and Büsch) evaluation of anti-doping education (Blank et al.), antecedents of doping attitude (García-Grimau et al.) and analgesics use (Melzer et al.).

Despite the offer for a protected space for underrepresented women researchers, the pool of authors in this special issue seems to feature established women researchers and less prolific or impactful male researchers (see: https://public.flourish.studio/visualisation/14246812/ and Supplementary Material). This could reflect the possibility that established women researchers tend to be supportive and nurturing of younger researchers, who still tend to be male. Notably, established male researchers publishing with a female researcher as first or corresponding author is missing from the collection. This might have been a consequence of how the themed collection was promoted. Submission was open to anyone (providing that the authorial team met the requirements for this collection) but female researchers were directly contacted and invited to submit an article, which might have skewed this outcome. Nonetheless, networking and collaboration deserve attention because there is a careful balance for women researchers to aim at. On the one hand, inter-gender collaboration may benefit male researchers more than females, especially if the male authors are at high academic level (26), but on the other hand, outputs with male authors being either first or corresponding author are more likely to describe the results in positive terms, which in turn leads to higher downstream citations (15).

The overall picture from this collection gives reassurance that women researchers do well in anti-doping sciences compared to other fields but it also raises forward-looking questions of how research collaboration can be encouraged to benefit from the unique contribution women can bring to advance the field. A further challenge is how underrepresented female researchers can be supported in closing the gender gap if not by offering a protected space where competition is limited to other female researchers. Following Li et al. (27) recommendations for mentoring, perhaps we also need to find ways to incentivise established ‘star’ female researchers to work with and mentor emerging, early career female researchers for scientific communication and impact.
