# Supplementary material for: Editorial: Women in anti-doping sciences & integrity in sport: 2021/22
Source: Front Sports Act Living. 2023 Jul 11;5:1248720. doi: 10.3389/fspor.2023.1248720 (PMC10368458; doi:10.3389/fspor.2023.1248720)
Supplement: Supplementary file 1 [file Datasheet1.pdf]

Supplementary material for "Editorial: Women in Anti-Doping Sciences & Integrity in Sport) by *Andrea Petróczi, Kim Nolte, Angela Schneider*

Table 1: Author profiles in the 'Women in Anti-Doping Science and Integrity in Sport: 2021-22 topic' (based on Web of Science, 2022 December)

Name	Gender	Active period	Number of outputs	Average number of citations per output	H-index
Bernier, N.	M	2022	2	1.0	1
Bhuiyan, H	M	-	-	-	-
Blank, C.	F	2009-2022	40	14.63	13
Börjesson, A	F	2016-2022	7	11.29	4
Buisson, C.	F	1999-2022	33	8.64	10
Büsch, D.	M	2004-2022	48	11.29	13
Butcher, L. H.G.	M	2020 2022	2	1	1
Casado, A.	M	2017-2022	36	5.0	7
Collomp, K.	F	1990-2022	88	19.73	23
De la Vega, R.	M	2021-2022	11	2.0	3
Ekström, L.	F	2007-2022	90	16.82	23
Elbe, A-M.	F	2005-2022	73	11.73	16
Erickson, M.	M	-	-	--	-
Garcia-Grimau, E.	F	2021-2022	4	1.25	1
Gatterer, K.	F	2018-2022	5	5.2	2
Kiss, A.	F	2017-2022	26	5.69	8
Lakner, Z.	M	1994-2022	114	10.88	15
Lehtihet, M.	M	2007-2022	33	13.61	14
Manges, T.	F	2021-2022	3	4.33	1
Melzer, M.	M	2010-2022	4	7.0	2
Oldham, A. C.	M	-2020-2022	3	1	1
Overbye, M.	F	2013-2022	11	10.09	6
Petróczi, A.	F	2007 - 2022	127	23.46	28
Pohanka, A.	M	2004-2022	53	17.06	19
Pöppel, K.	F	2016-2022	4	3.0	3
Schlosberger, W.	M	2007-2022	71	13.08	16
Schneider, A.	F	1984-2022	43	14.21	12
Schüler, T.	M	2022	1	-	-
Seidel, K.	M	2022	1	-	-
Soós, S.	M	1989-2022	35	6.54	7
Stephanou, C.	F	2022	2	0.50	1
Strahler, K.	F	-	-	-	-
Streicher, B.	M	2017-2022	16	21.63	9
Teetzel, S.	F	2012-2022	15	4.93	5
Walter, N.	F	2016-2022	11	6.36	4

Table 2: Gender differences in academic performance indicators (based on data presented in Table 1; values in square brackets are with zero inputted for no record)

	**Male**	**Female**
Active years	12.2 [10.7]	12.7 [12.3]
Average number of outputs	35.0 [29.7]	34.7 [29.6]
Average number of citations per output	10.0 [7.9]	10.0 [7.9]
Average H-index	9.9 [7.9]	9.8 [7.8]
